# Case Report: Dupilumab as a steroid-sparing therapeutic option in refractory anti-p200 pemphigoid: real-world effectiveness in a patient with significant comorbidities

**DOI:** 10.3389/fimmu.2026.1922276

**Published:** 2026-09-07

**Authors:** Aikaterini Kyriakou, Christos Minas, Antonios Fantakis, Athanasia Tsaousi, Anastasia Tsitlakidou, Parthena Meltzanidou, Anthi-Maria Lazaridi, Alexandra Fleva, Anastasia Giannakou, Elisavet Lazaridou, Aikaterini Patsatsi

**Affiliations:** 1 B Dermatology Clinic, Aristotle University School of Medicine, Papageorgiou General Hospital, Thessaloniki, Greece; 2 Center of Expertise on AIBD, B Dermatology Department, Aristotle University School of Medicine, Papageorgiou General Hospital, Thessaloniki, Greece; 3 Aristotle University of Thessaloniki, Thessaloniki, Greece; 4 1^st^ Surgical Department, Aristotle University School of Medicine, Papageorgiou General Hospital, Thessaloniki, Greece; 5 Center of Expertise on AIBD, Department of Immunology - Histocompatibility, Papageorgiou General Hospital, Thessaloniki, Greece

**Keywords:** anti-laminin γ1 pemphigoid, anti-p200 pemphigoid, autoimmune blistering disease, dupilumab, real-world evidence, steroid-sparing therapy

## Abstract

Anti-p200 pemphigoid is a rare autoimmune subepidermal blistering disease with limited evidence guiding its management. We report a 49-year-old woman with refractory anti-p200 pemphigoid who failed treatment with high-dose prednisone and dapsone and developed significant corticosteroid-related toxicity, including hypertension, hypertrichosis, steroid-induced myopathy, and psychological distress. Given her history of complicated diverticulitis requiring bowel resection, dupilumab was initiated as a steroid-sparing therapeutic option. Pruritus improved within two weeks, near-complete remission was achieved after two months, and sustained disease control was maintained for six months, allowing substantial corticosteroid tapering without treatment-related adverse events.

## 1 Introduction

Anti-p200 pemphigoid, also known as anti-laminin γ1 pemphigoid, is a rare autoimmune subepidermal blistering disease characterized by autoantibodies directed against a 200-kDa protein located within the basement membrane zone, most commonly laminin γ1 (1, 2). Clinically, patients typically present tense bullae, vesicles, erosions, and pruritus, often resembling bullous pemphigoid and other subepidermal autoimmune blistering diseases (2).

Diagnosis relies on the integration of clinical findings, histopathology, direct immunofluorescence, and the detection of anti-p200/anti-laminin γ1 autoantibodies by specialized immunological assays (2). Histopathological examination usually demonstrates a subepidermal blister with neutrophilic and eosinophilic inflammatory infiltrates, whereas direct immunofluorescence reveals linear deposition of IgG and C3 along the basement membrane zone (2).

Evidence-based therapeutic recommendations remain unavailable. Systemic corticosteroids constitute the cornerstone of treatment and are frequently combined with immunomodulatory agents such as dapsone. Nevertheless, relapses are common and prolonged corticosteroid exposure is often associated with substantial treatment-related morbidity (3).

Herein, we report a case of refractory anti-p200 pemphigoid successfully treated with dupilumab after failure of systemic corticosteroids and dapsone. The case highlights the real-world effectiveness of dupilumab as a steroid-sparing therapeutic option in a patient with significant corticosteroid-related toxicity and comorbid diverticulitis requiring surgical management.

## 2 Case presentation

A 49-year-old woman was referred to our tertiary dermatology outpatient clinic with a six-month history of an autoimmune blistering disorder initially diagnosed as bullous pemphigoid, based only on clinical features. Before referral, she had been treated with oral prednisone 60 mg/day (approximately 1 mg/kg/day) for four months without achieving satisfactory disease control.

Clinical examination revealed widespread post-inflammatory hyperpigmentation involving the trunk and extremities, accompanied by multiple tense bullae and vesicles predominantly affecting the chest, neck, upper limbs, and lower limbs. Numerous small vesicles were observed arising on pre-existing hyperpigmented areas of the trunk. The patient reported severe pruritus. No mucosal involvement was detected.

Her medical history was notable for complicated diverticulitis requiring bowel resection and colostomy formation. Additional surgical intervention was being considered at the time of presentation. Prolonged systemic corticosteroid therapy had already resulted in hypertension, hypertrichosis, steroid-induced myopathy, and significant psychological distress.

Laboratory evaluation demonstrated mild peripheral eosinophilia (absolute eosinophil count: 1.32K/μL). Histopathological examination revealed a subepidermal blister containing a mixed inflammatory infiltrate composed predominantly of neutrophils and eosinophils, with scattered lymphocytes. Mild-to-moderate diffuse inflammatory infiltrates consisting of similar inflammatory cells were present in the underlying dermis.

Direct immunofluorescence demonstrated linear deposition of IgG and C3 along the basement membrane zone, without IgA deposition. Serological testing for BP180 and BP230 autoantibodies was negative. Indirect immunofluorescence using a BIOCHIP mosaic demonstrated a faint linear deposition of IgG along the dermal side (floor) of salt-split skin. Additional testing showed positivity for laminin β4, while BP180, BP230, laminin-332, collagen VII, desmoglein 1, desmoglein 3, and envoplakin antibodies were negative. Anti-laminin γ1 (p200) reactivity was considered positive (titer >1:10), confirming the diagnosis of anti-p200 pemphigoid.

Prednisone was continued at 30 mg/day (approximately 0.5 mg/kg/day) when dapsone 50 mg/day was initiated. Despite three months of treatment, the patient continued to develop new bullous lesions and experienced persistent pruritus, indicating an inadequate therapeutic response.

Given the refractory disease course, corticosteroid-related adverse effects, and the need to avoid prolonged escalation of immunosuppression because of her gastrointestinal comorbidity and anticipated surgical management, dupilumab was initiated at a loading dose of 600 mg followed by 300 mg every two weeks.

**Figure 1:**
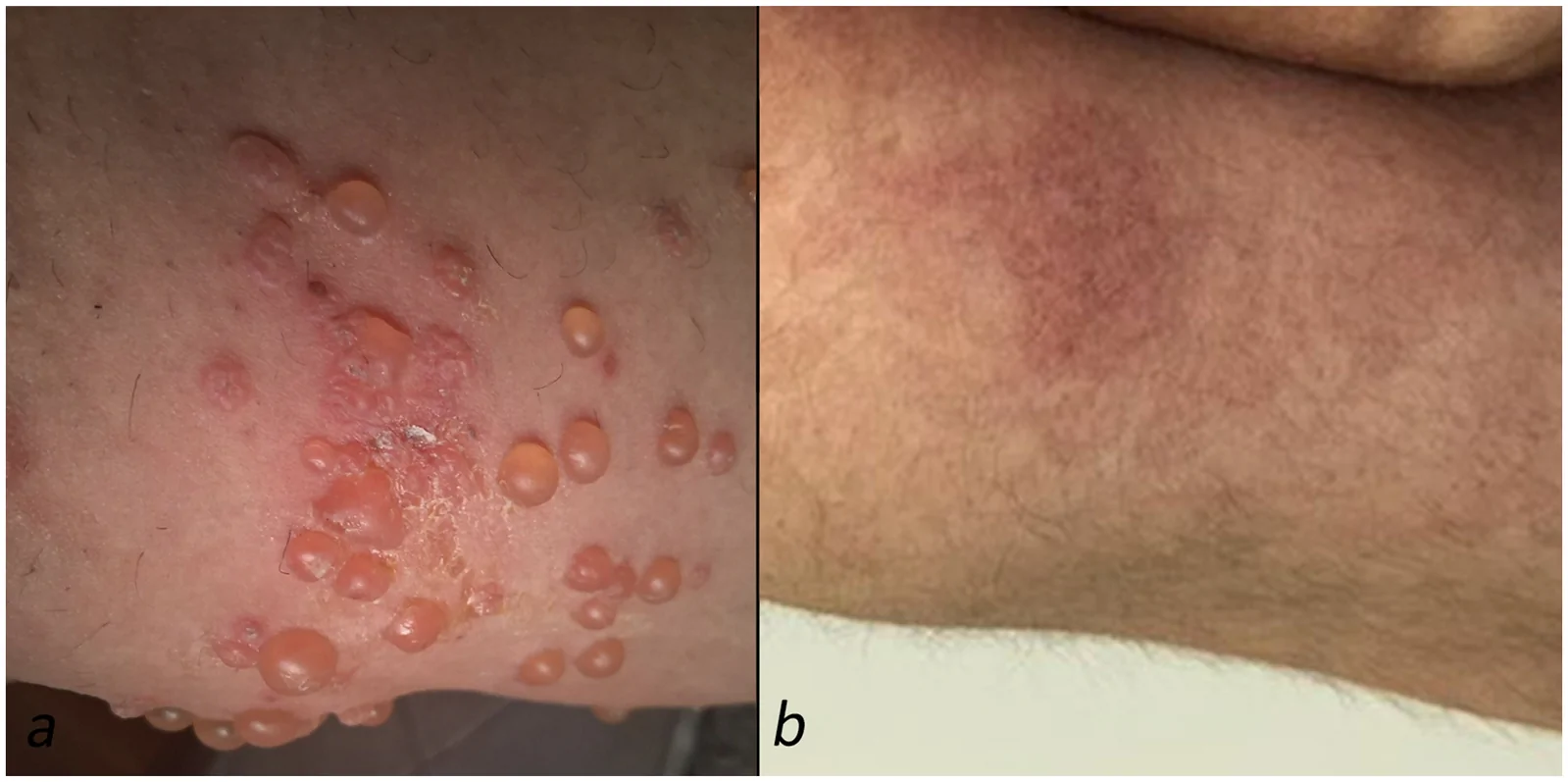
Patient’s left arm, **(a)** before treatment, and **(b)** after treatment.

**Figure 2:**
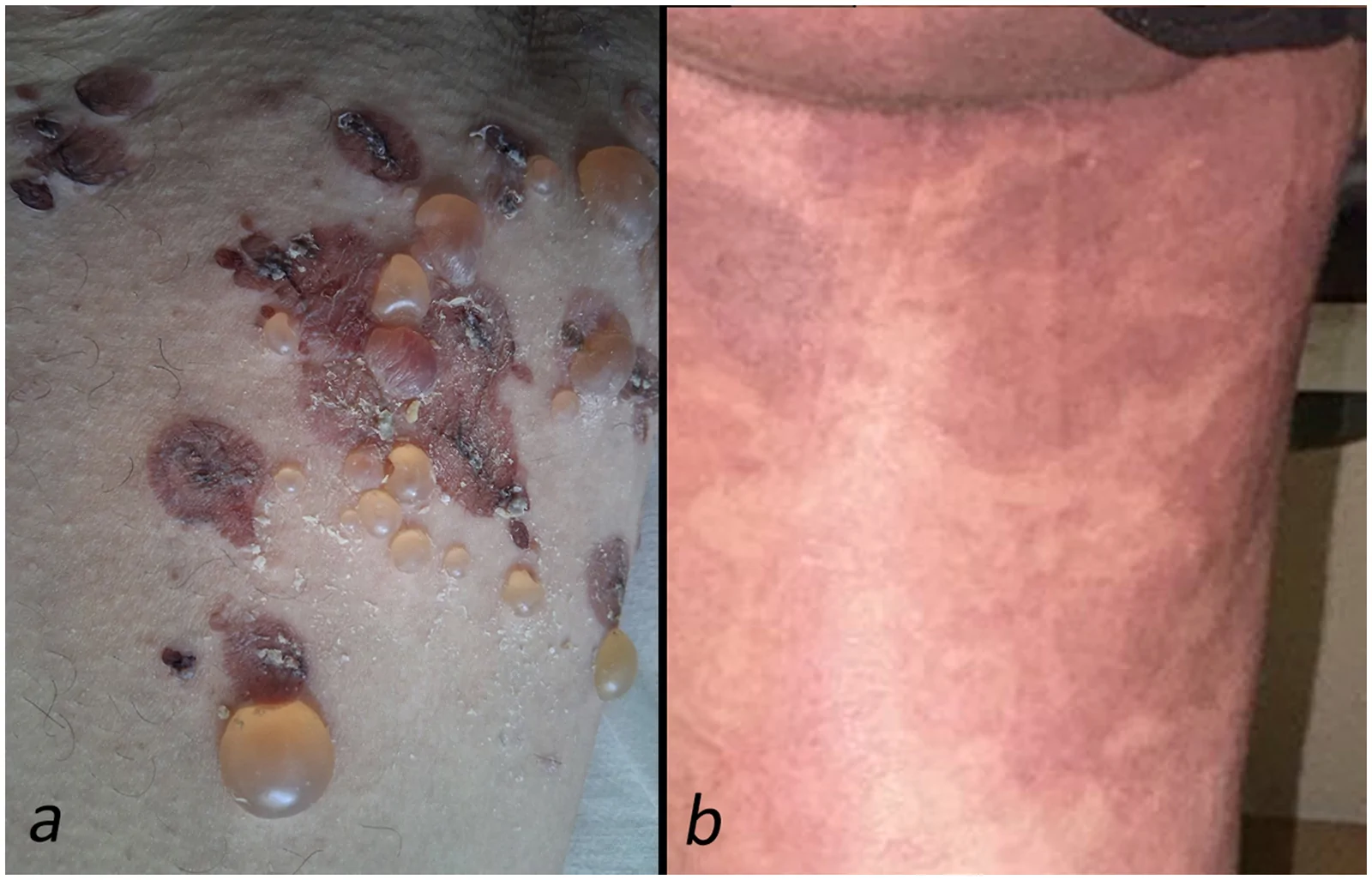
Patient’s left gluteal region, **(a)** before treatment, and **(b)** after treatment.

**Figure 3:**
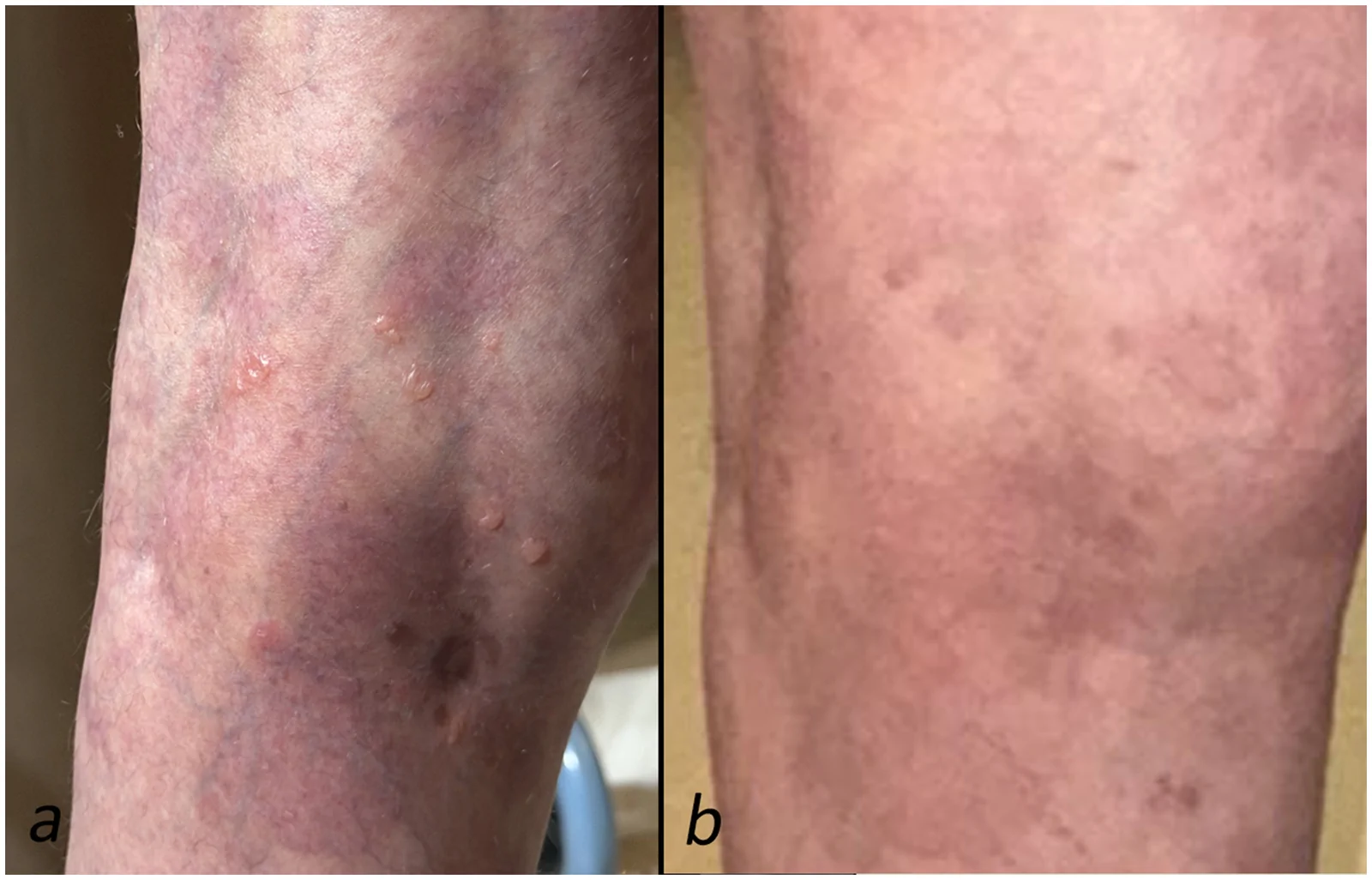
Patient’s left popliteal region, **(a)** before treatment, and **(b)** after treatment.

**Figure 4:**
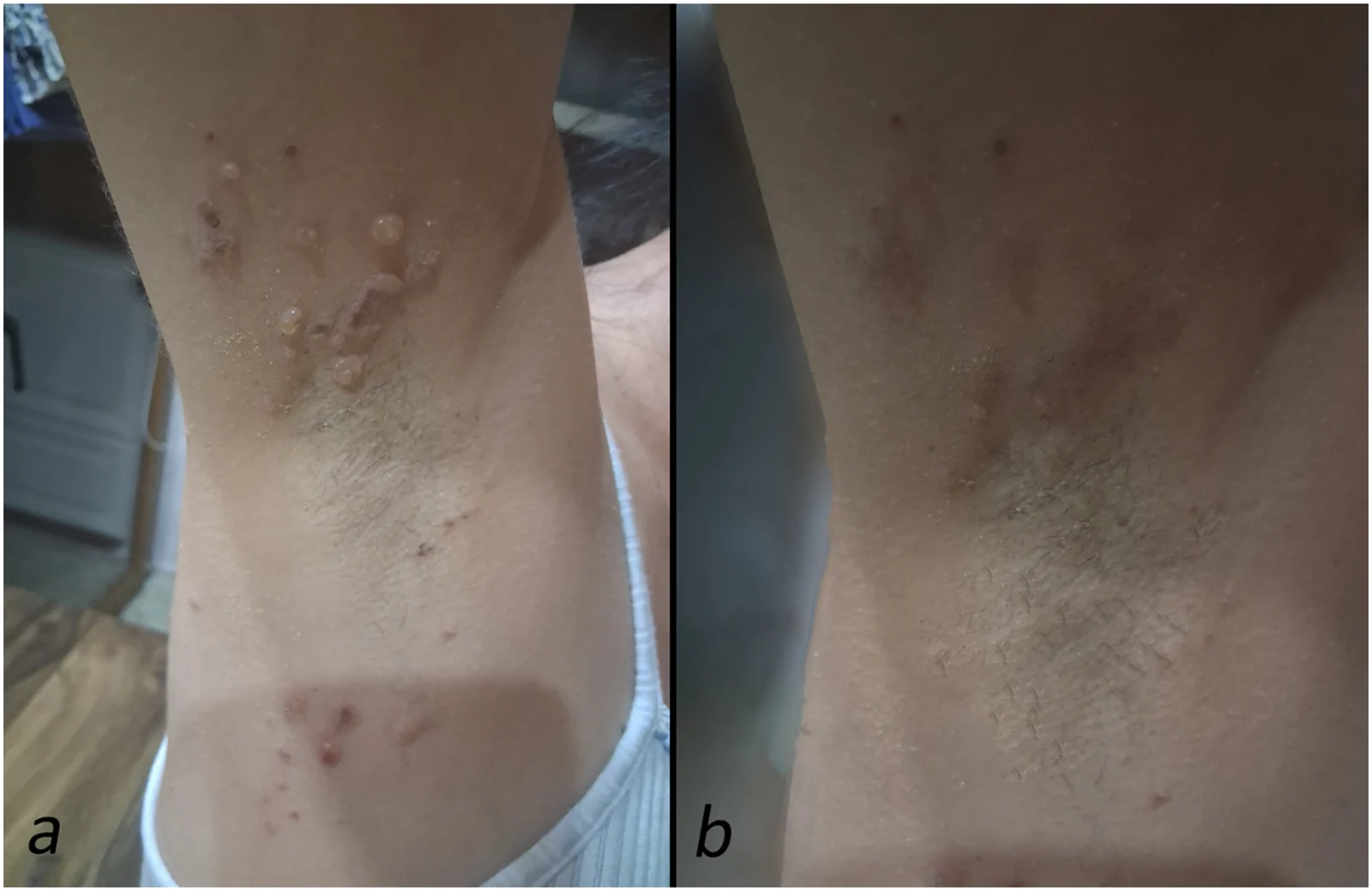
Patient’s right axilla, **(a)** before treatment, and **(b)** after treatment.

Baseline pruritus severity was 8/10 on the Numeric Rating Scale (NRS). Two weeks after initiation of dupilumab, the NRS score had improved to 2/10, accompanied by marked clinical improvement. After one month, a marked reduction in blister formation and inflammatory activity was observed. Two months after starting dupilumab, complete resolution of active disease was achieved except for a single residual lesion on the ankle. Residual post-inflammatory hyperpigmentation persisted, but no new bullae or vesicles developed (**Figures 1**–**4**).

At six-month follow-up, the patient remained in sustained clinical remission. Dapsone had been discontinued, and prednisone had been successfully tapered from 60 mg/day to 2.5 mg/day without disease recurrence. No adverse events related to dupilumab were observed during the six-month follow-up period.

## 3 Discussion

Anti-p200 pemphigoid remains a therapeutic challenge because of the absence of standardized treatment guidelines. According to the largest systematic review currently available, systemic corticosteroids, either alone or in combination with immunomodulatory agents, remain the most frequently employed treatment strategy, while dapsone is the most used adjunctive therapy (3, 4). Although many patients eventually achieve disease control, relapses are frequent and prolonged treatment often results in significant adverse effects (3).

The present patient represented a particularly challenging real-world therapeutic scenario. Despite treatment with high-dose prednisone and a subsequent three-month course of dapsone, disease activity persisted with ongoing blister formation and severe pruritus. Moreover, prolonged corticosteroid treatment had already resulted in hypertension, hypertrichosis, steroid-induced myopathy, and considerable psychological distress. Therapeutic decision-making was further complicated by the patient’s history of complicated diverticulitis requiring bowel resection and colostomy formation, together with the possibility of future surgical intervention. In this setting, escalation of conventional immunosuppressive therapy was considered undesirable, creating a need for an effective steroid-sparing strategy.

Over the last decade, several targeted therapeutic approaches have been reported in isolated cases of anti-p200 pemphigoid. Successful outcomes have been described with rituximab (5), ustekinumab (6), apremilast (7), guselkumab (8), tofacitinib (9), methotrexate-based treatment (10), and, most recently, stapokibart combined with corticosteroids (11). Furthermore, omalizumab has demonstrated efficacy in a patient with refractory bullous pemphigoid associated with anti-p200 antibodies (12). Collectively, these observations suggest that targeted immune modulation may represent a promising therapeutic strategy in patients with refractory disease; however, evidence remains limited to isolated case reports and small case series.

Dupilumab is a fully human monoclonal antibody targeting the interleukin-4 receptor alpha subunit, thereby inhibiting both IL-4 and IL-13 signaling pathways. Increasing evidence supports its efficacy in bullous pemphigoid and other inflammatory dermatoses, particularly regarding rapid pruritus control and reduction of corticosteroid requirements. Although the precise role of type 2 inflammation in anti-p200 pemphigoid remains incompletely understood, the rapid improvement of pruritus observed in our patient suggests that IL-4/IL-13 signaling may contribute to disease activity in at least a subset of patients.

The clinical response observed in our patient was both rapid and sustained. Pruritus improved within two weeks of treatment initiation, substantial regression of lesions occurred after one month, and near-complete remission was achieved after two months (**Figures 1**-**4**). Importantly, disease control was maintained throughout six months of follow-up, allowing discontinuation of dapsone and tapering of prednisone from 60 mg/day to 2.5 mg/day without relapse. No treatment-related adverse events were observed. These findings provide real-world evidence supporting the effectiveness and safety of dupilumab in a patient who would likely have been underrepresented in conventional clinical trials owing to significant comorbidities and the need for surgical management.

Although conclusions cannot be drawn from a single case, our experience suggests that dupilumab may represent a valuable steroid-sparing therapeutic option in selected patients with anti-p200 pemphigoid, particularly when conventional therapies are ineffective, poorly tolerated, or contraindicated. Further reports and collaborative studies are needed to clarify the role of IL-4/IL-13 blockade in the therapeutic armamentarium of anti-p200 pemphigoid.

## 4 Conclusion

This case demonstrates successful off-label treatment of refractory anti-p200 pemphigoid with dupilumab in a patient with significant corticosteroid toxicity and comorbid diverticulitis requiring surgical management. Dupilumab was associated with rapid improvement of pruritus, near-complete remission within two months, sustained disease control during six months of follow-up, and substantial corticosteroid reduction from 60 mg/day to 2.5 mg/day. These findings contribute real-world evidence supporting dupilumab as a potential steroid-sparing therapeutic option in selected patients with anti-p200 pemphigoid.

## Data Availability

The original contributions presented in the study are included in the article/supplementary material. Further inquiries can be directed to the corresponding author.
